# MDCT Findings in Gastrointestinal Perforations and the Predictive Value according to the Site of Perforation

**DOI:** 10.3390/tomography8020056

**Published:** 2022-03-03

**Authors:** Stefania Romano, Carmela Somma, Antonio Sciuto, Warissara Jutidamrongphan, Daniela Pacella, Francesco Esposito, Marta Puglia, Claudio Mauriello, Khanin Khanungwanitkul, Felice Pirozzi

**Affiliations:** 1 Department of Radiology, “S. Maria delle Grazie” Hospital, 80078 Pozzuoli, Italy; marta.puglia@aslnapoli2nord.it; 2 Department of Neuroradiology, “Umberto I” Hospital, 80131 Nocera, Italy; csomma89@gmail.com; 3 Department of Surgery, “S. Maria delle Grazie” Hospital, 80078 Pozzuoli, Italy; antonio.sciuto@aslnapoli2nord.it (A.S.); francesco.esposito1@aslnapoli2nord.it (F.E.); claudio.mauriello@aslnapoli2nord.it (C.M.); felice.pirozzi@aslnapoli2nord.it (F.P.); 4 Department of Radiology, Faculty of Medicine, Prince of Songkla University, Hat Yai, Songkhla 90110, Thailand; warissara.jut@gmail.com (W.J.); khanin14@gmail.com (K.K.); 5 Department of Public Health, Federico II University of Naples, 80131 Naples, Italy; daniela.pacella@unina.it

**Keywords:** MDCT, intestinal perforations, pneumoperitoneum, acute abdomen, surgery, emergency

## Abstract

*Background:* Gastrointestinal perforations are a frequent cause of acute abdominal symptomatology for patients in the emergency department. The aim of this study was to investigate the findings of multidetector-row computed tomography of gastrointestinal perforations and analyze the impact of any imaging signs on the presurgical identification of the perforation site. *Methods:* We retrospectively reviewed emergency MDCT findings of 93 patients submitted to surgery for gastrointestinal perforation at two different institutions. Two radiologists separately reviewed the emergency MDCT examinations performed on each patient, before and after knowing the surgical diagnosis of the perforation site. A list of findings was considered. Positive predictive values were estimated for each finding with respect to each perforation site, and correspondence analysis (CA) was used to investigate the relationship between the findings and each of the perforation types. *Results:* We did not find inframesocolic free air in sigmoid colorectal perforations, and in rare cases, only supramesocolic free fluid in gastroduodenal perforations was found. A high PPV of perivisceral fat stranding due to colonic perforation and general distension of upstream loops and collapse of downstream loops were evident in most patients. *Conclusions:* Our data could offer additional information on the perforation site in the case of doubtful findings to support surgeons, especially in planning a laparoscopic approach.

## 1. Introduction

Gastrointestinal (GI) perforations are common surgical emergencies, accounting for approximately 3% of acute abdomen syndrome cases [1]. They consist of discontinuities of the GI wall that allow the intestinal lumen and the extraluminal space to communicate. Breaches can appear due to different causes, such as peptic ulcers, inflammatory bowel disease, blunt or penetrating trauma, iatrogenic factors, foreign bodies or neoplasms [2,3,4,5]. Early diagnosis of a GI tract perforation, together with identification of the site and cause, can facilitate treatment and improve prognosis, having a great impact on therapeutic management, including the type of surgery or a focused conservative choice [6]. Clinical diagnosis of the exact site of GI tract perforation may be difficult, as the clinical signs and symptoms may be nonspecific. Consequently, the role of the emergency radiologist appears crucial, as the final diagnosis is mainly based on imaging results, particularly on CT [7]. Patients with acute abdominal pain and clinical suspicion of bowel perforation are usually first submitted to X-ray examination, and the direct radiographic sign of bowel perforation is represented by the evidence of free abdominal gas. However, to answer the surgical question regarding the site of perforation, CT examination is the most accurate tool to allow a diagnosis [8]. The predictive value of MDCT to detect the site of perforation has been already investigated by several authors and the results of studies already published in the radiologic literature: distribution of free air, concentration of extraluminal air bubbles, segmental bowel wall thickening, focal defect of the bowel wall, etc. [9,10]. More recently, predictors of perforation at different GI tract sites have been reported: focal bowel wall discontinuity for the stomach, duodenal bulb and left colon; mottled extraluminal air bubbles for the retroperitoneal duodenum and right colon; and segmental bowel wall thickening for the small bowel [11]. However, in radiological clinical practice in the emergency department, sometimes, correlating imaging findings with a suggestive site of perforation may not be easy, and data from the literature are not always applicable.

The aim of this study was to investigate the MDCT findings of patients who underwent emergency surgery for gastrointestinal perforations and re-evaluate them according to the site of lesion in order to analyze the impact of imaging signs on the presurgical identification of the perforation site.

## 2. Materials and Methods

A retrospective evaluation of the surgical database of adult patients who presented at the emergency department between January 2017 and August 2019 at two different institutions (Santa Maria delle Grazie Hospital in Pozzuoli, Italy, and Songklanagarind Hospital in Songkhla, Thailand) with acute abdominal symptomatology and submitted to surgery for gastrointestinal tract perforation was performed. As this was a retrospective study with anonymization of all personal patient data included in the review process, no local Ethical Committee permission was required in our institutions. We retrospectively reviewed the emergency MDCT imaging findings of 93 adult patients: 58 from Santa Maria delle Grazie Hospital and 35 from Songklanagarind Hospital (59 men (63.4%) and 34 women (36.5%) with a mean age of 66.7 years old)), with final diagnosis of gastrointestinal perforation and submitted to surgery within 12 h. CT scans of the abdomen were performed using one of the following scanners available at the two institutions: Dual Source DECT 128 detector row scanner (Somatom Drive, Siemens Healhineers), two 64 MDCT scanners (Lightspeed GE; Ingenuity, Philips) at Santa Maria delle Grazie Hospital, 160 detector rows MDCT (Aquilion Prime, Toshiba) and DECT 512 detector rows scanners (Revolution, GE) at Songklanagarind Hospital.

Due to the different locations of the two institutions involved, as well as the retrospective nature of the study, the imaging methodology was heterogeneous; however, all patients underwent a CT scan in the supine position starting from the diaphragm down to the pubic symphysis (slice thickness of 2.5/3 mm, back reconstructions at less than 1 mm). Intravenous contrast medium was administered in all cases and oral contrast material in one patient. A precontrast abdominal scan was performed in all examinations on the single energy machines, whereas patients imaged with DE equipment underwent postcontrast scanning only. A contrast-enhanced phase CT was performed in most cases with a 55 s delay (90–100 mL of iomeprol, infusion rate at 3 mL/s), followed by 30 mL of saline flush at the same injection rate. The images were retrospectively reviewed on dedicated workstations.

Two radiologists with different degrees of experience in abdominal and emergency radiology (one with 19 years’ experience, one final-year resident at Santa Maria delle Grazie Hospital, one with 4 years’ experience and one final-year resident at Songklanagarind Hospital) separately re-evaluated the axial and multiplanar reconstructions of the anonymized CT examinations using lung and soft tissue level window visualization. Both reviewers were blinded to any patient medical records but were informed that all cases had a surgical diagnosis of GI perforation.

In the analysis of each CT scan, the following CT findings were evaluated before knowing the surgical diagnosis of the perforation site: intra- and retroperitoneal free air and its location; presence and distribution of air bubbles; free fluid; fluid collections; focal defect in the bowel wall, if visible; air/fluid intestinal loop distension; presence of collapsed loops; evidence of segmental or diffuse abnormal bowel wall thickening and enhancement.

After consulting the surgical diagnosis, a consensual re-evaluation of all CT exams and findings was performed, also considering the surgical point of perforation, in order to attest the features of GI segments located, respectively, upstream and downstream to the perforation site (with attention to the lumen, wall thickness and enhancement); appearance of the GI segment involved in perforation (with attention to the lumen, wall thickness and enhancement); presence of perivisceral fat stranding; and presence of perivisceral fluid collection.

All data were collected with Excel 15.34 (© 2022 Microsoft Corporation, Washington, WA, USA).

Data are reported as frequency (percentages). The *p*-value on the contingency table was computed with the chi-square test. Positive predictive values were estimated for each finding with respect to each perforation type. False positives for each perforation site were considered as all the cases where the finding was present in the other sites of perforation. Correspondence analysis (CA) was used to investigate the relationship between the findings and each perforation type. Hierarchical clustering was performed on the CA dimensions to identify the clusters of associated findings. All analyses were performed using the statistical computing software R version 4.0.2.

## 3. Results

A review of the surgical database showed the following perforation sites: cecal appendix (3 cases), ascending colon (2 cases), cecum (2 cases), jejunum (5 cases), descending colon (5 cases), duodenum (13 cases), ileum (13 cases), rectum (4 cases), sigmoid colon (24 cases), stomach (18 cases) and transverse colon (4 cases).

We divided these sites of perforation into four groups to easily evaluate the predictive value of each CT sign: stomach and duodenum (Group A), small bowel loops (Group B), colon from the cecum to the descending colon (Group C) and sigmoid colon and rectum (group D).

All findings for each group are schematized in Table 1 and Table 2.

**Figure 1:**
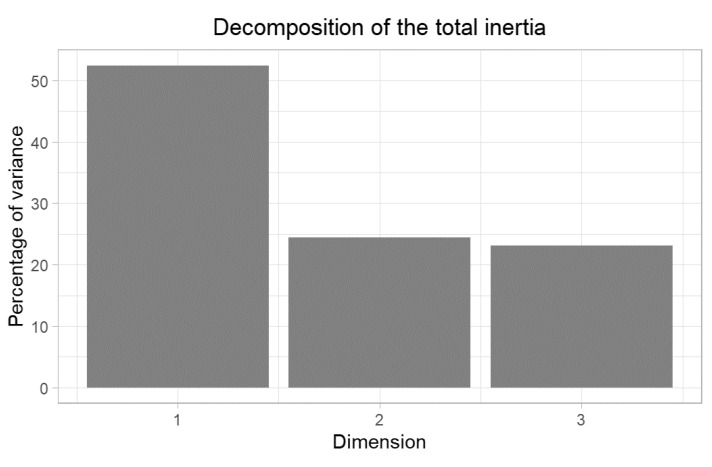
Scree plot showing the dimensions extracted from the CA and their explained variance.

The plot in Figure 1 also displays the findings in a concise view. The chi-square statistics on the contingency table show a significant association between the findings and the perforation site (*p* < 0.001). To better investigate the association, correspondence analysis was performed, and the results on the dataset, which has no missing values, are shown below. Figure 1 shows the scree plot of the CA. The first two dimensions express 76.93% of the total dataset inertia. The first factor is major: it expresses 52.49% of the data variability. An estimation of the right number of axes to interpret suggests restricting the analysis to the description of the first axis. This axis presents an amount of inertia greater than those obtained by the 0.95 quantile of random distributions (52.49% against 52.12%).

**Figure 2:**
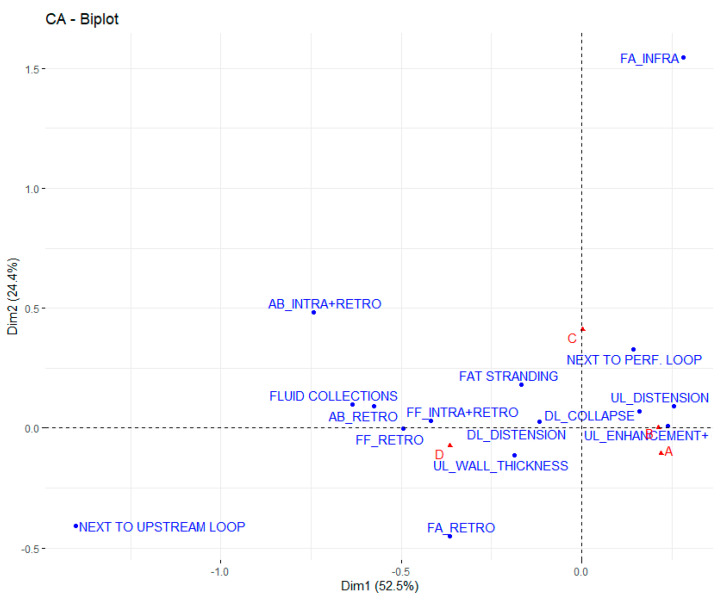
Biplot of spatial relationships across dimensions for each perforation site and finding.

The biplot in Figure 2 shows the spatial relationship across the CA dimensions among findings and perforation sites.

Dimension 1 shows factors such as FAT STRANDING, FS NEXT TO PERF. LOOP, UL_DISTENSION, FF_SUPRA+INFRA and WALL ENHANCEMENT (to the right of the graph, characterized by a strongly positive coordinate on the axis) opposed to factors such as NEXT TO UPSTREAM LOOP, FLUID COLLECTIONS, FF_RETRO, AB_RETRO, FA_RETRO, AB_INTRA+RETRO and FA_INTRA+RETRO (to the left of the graph, characterized by a strongly negative coordinate on the axis).

The findings AB_RETRO, FF_RETRO, FF_INTRA+RETRO, FS NEXT TO UPSTREAM LOOP, FLUID COLLECTIONS, UL_ENHANCEMENT are highly correlated with the dimension (respective correlation of 0.96, 0.99, 0.97, 0.92, 0.97, 0.94 and 0.96), and Dimension 1 can be considered as being mainly explained by them.

**Figure 3:**
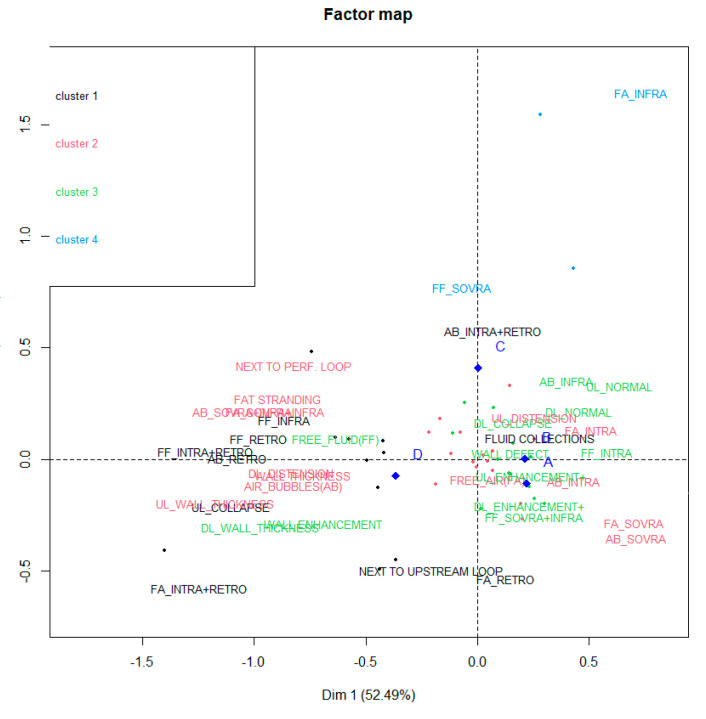
Cluster analysis performed on the results.

A hierarchical cluster (HC) analysis was also performed on the coordinates of the findings and perforation sites extracted from the CA. The HC revealed four clusters, as in Figure 3.

Cluster 1 is mainly identified by findings FS NEXT TO UPSTREAM LOOP, AB_INTRA+RETRO, FLUID COLLECTIONS, AB_RETRO, FF_RETRO, UL_COLLAPSE, FA_INTRA+RETRO and FA_RETRO. The cluster is characterized by significant:

- High occurrence of perforation site D;
- Low occurrence of perforation sites A and B.

Cluster 2, instead, is mainly identified by findings FAT STRANDING, FS NEXT TO PERF. LOOP, FA_SUPRA, AB_SUPRA and UL_DISTENSION. It is characterized by significant:

- High occurrence of perforation site A;
- Low occurrence of perforation site B.

Cluster 3 is identified by findings DL_WALL_THICKNESS, UL_NORMAL, FF_SUPRA+INFRA and WALL ENHANCEMENT and characterized by significant:

- High occurrence of perforation site A;
- Low occurrence of perforation site B.

Cluster 4 consists of the two outlier findings FA_INFRA and FF_SUPRA. This group is characterized by significant:

- High occurrence of perforation site C.

## 4. Discussion

**Figure 4:**
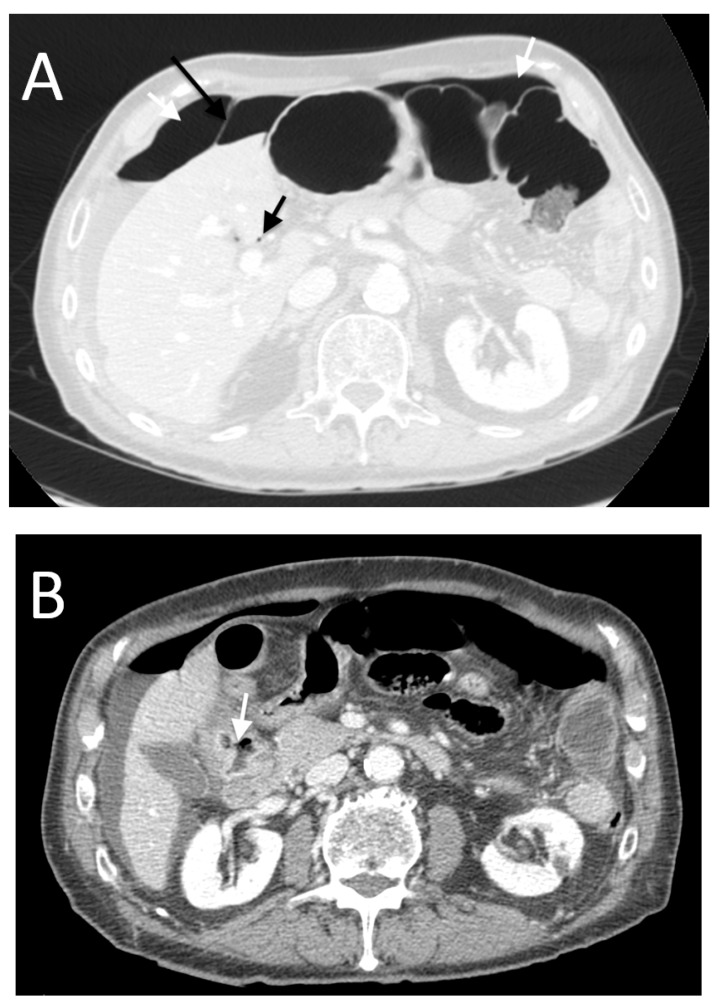
An 82-year-old male with perforation of the first portion of the duodenum caused by a peptic ulcer. The CT images show subdiaphragmatic free peritoneal air (white arrows in (**A**)) with “falciform ligament sign” (long black arrow in (**A**)). Note the periportal free air sign (short black arrow in (**A**)). In this case, we also found a focal wall defect (arrow in (**B**)).

For perforations of the stomach and first portion of the duodenum, we found free air in 87% of cases above all intraperitoneal supramesocolic located (Figure 4), with a PPV equal to 44%.

**Figure 5:**
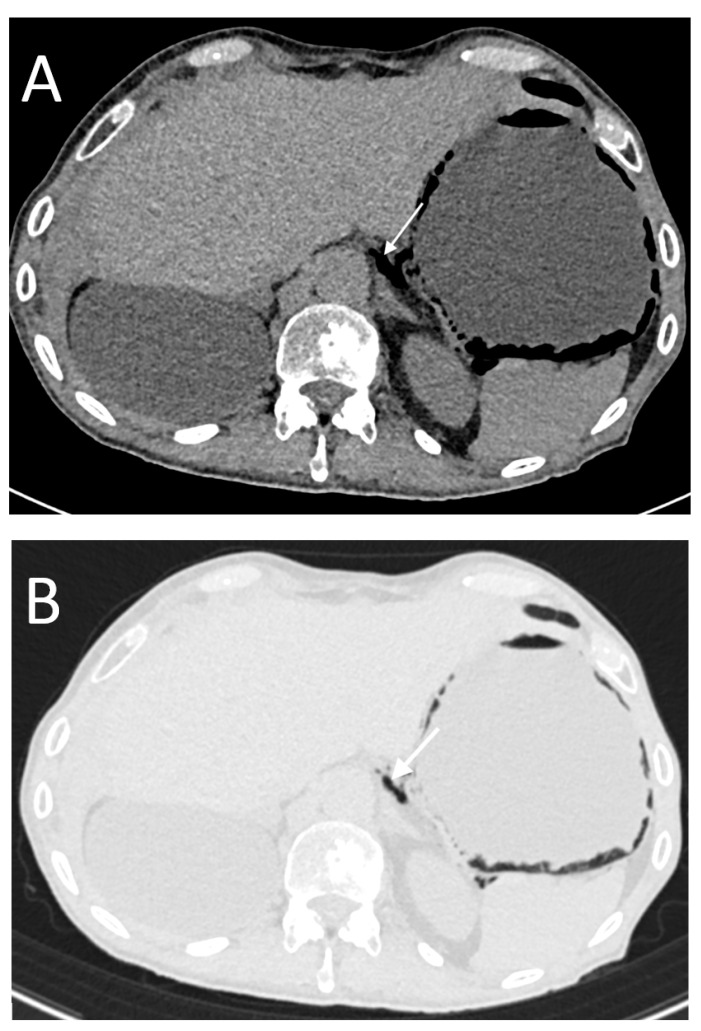
A 61-year-old male with perforation of the posterior wall of the stomach. The precontrast CT scan shows retroperitoneal free air (arrow in (**A**,**B**)) only, with no evidence of gastric wall breach. Note the diffuse parietal pneumatosis of the gastric wall.

These data are in agreement with data from the literature, in particular Yeung et al. in 2004 [9], Furukawa et al. in 2005 [12] and Cho et al. in 2009 [13], whose studies showed that in patients with perforation of the duodenal bulb or stomach, pneumoperitoneum is usually abundant, and it is noted around the liver and stomach, with frequent “falciform ligament sign” and “periportal free gas sign”. In 19% of gastroduodenal perforation cases, we found free retroperitoneal air (Figure 5) (PPV of 38%). According to the literature, perforation of the posterior wall of the stomach or the first portion of the duodenum can also cause free air in the lesser sac and also a walled-off or confined perforation [14].

**Figure 6:**
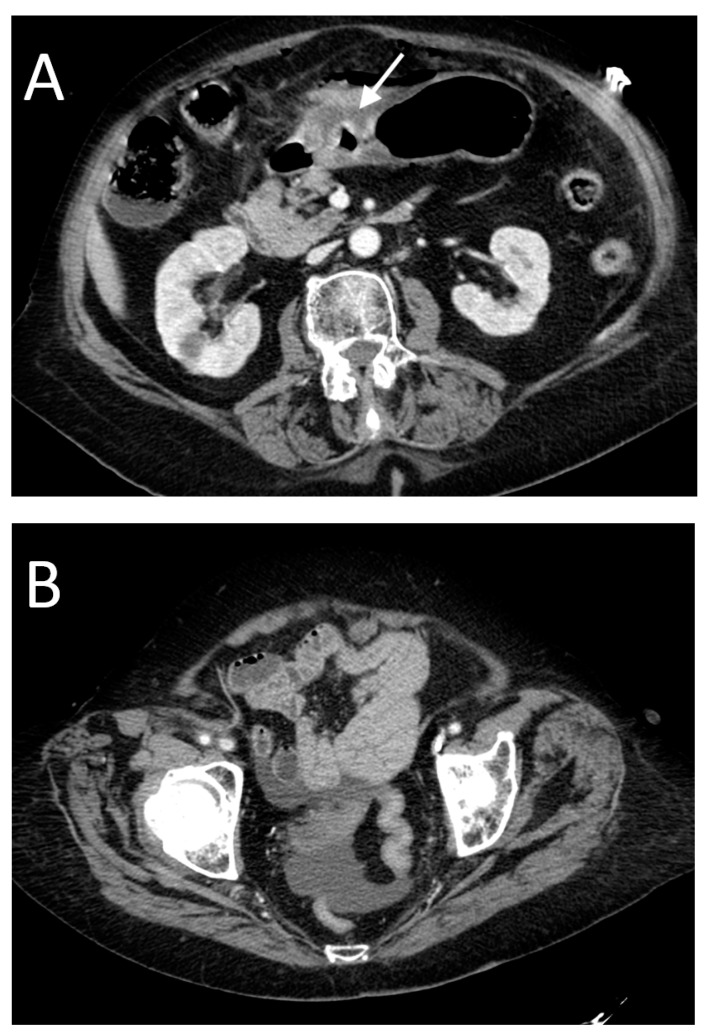
A 75-year-old female with perforation of the anterior gastric wall. In this case, CT shows focal abnormal wall thickening at the site of perforation (arrow in (**A**)), with the presence of a peripheral small amount of free air. There is also free fluid in the pelvis (**B**).

Moreover, extraluminal free air or gas bubbles located only in the retroperitoneal space, in particular in the right anterior pararenal space, has been reported as a reliable CT finding for diagnosing duodenal perforation beyond the bulbar segment, where gas typically outlines the lateral border of the psoas muscle [15]. Air bubbles close to the perforation site were found in 77% of patients in Group A, with a PPV equal to 41%. Regarding the evidence of free abdominal fluid, in our study, 84% of gastroduodenal perforation (PPV of 38%) was found, mainly intraperitoneal with evidence of retroperitoneal fluid in just 12% of cases (PPV of 23%). Free fluid was both supra and inframesocolic located in 85% of cases with a PPV of 46%, whereas only supramesocolic fluid was found in 8% of cases. This result differs somewhat from the study of Ghekiere in 2007 [16], reporting that supramesocolic free fluid between the head of the pancreas and duodenum had the highest PPV for gastroduodenal ulcer perforation. In gastroduodenal perforation, we found a higher PPV of segmental abnormal wall thickness (Figure 6) and segmental abnormal wall enhancement at the perforation site (PPVs of 38% and 46%, respectively) than the other three groups. This is an interesting finding, because few authors in the literature evaluated the predictive value of these findings according to the different perforation sites.

**Figure 7:**
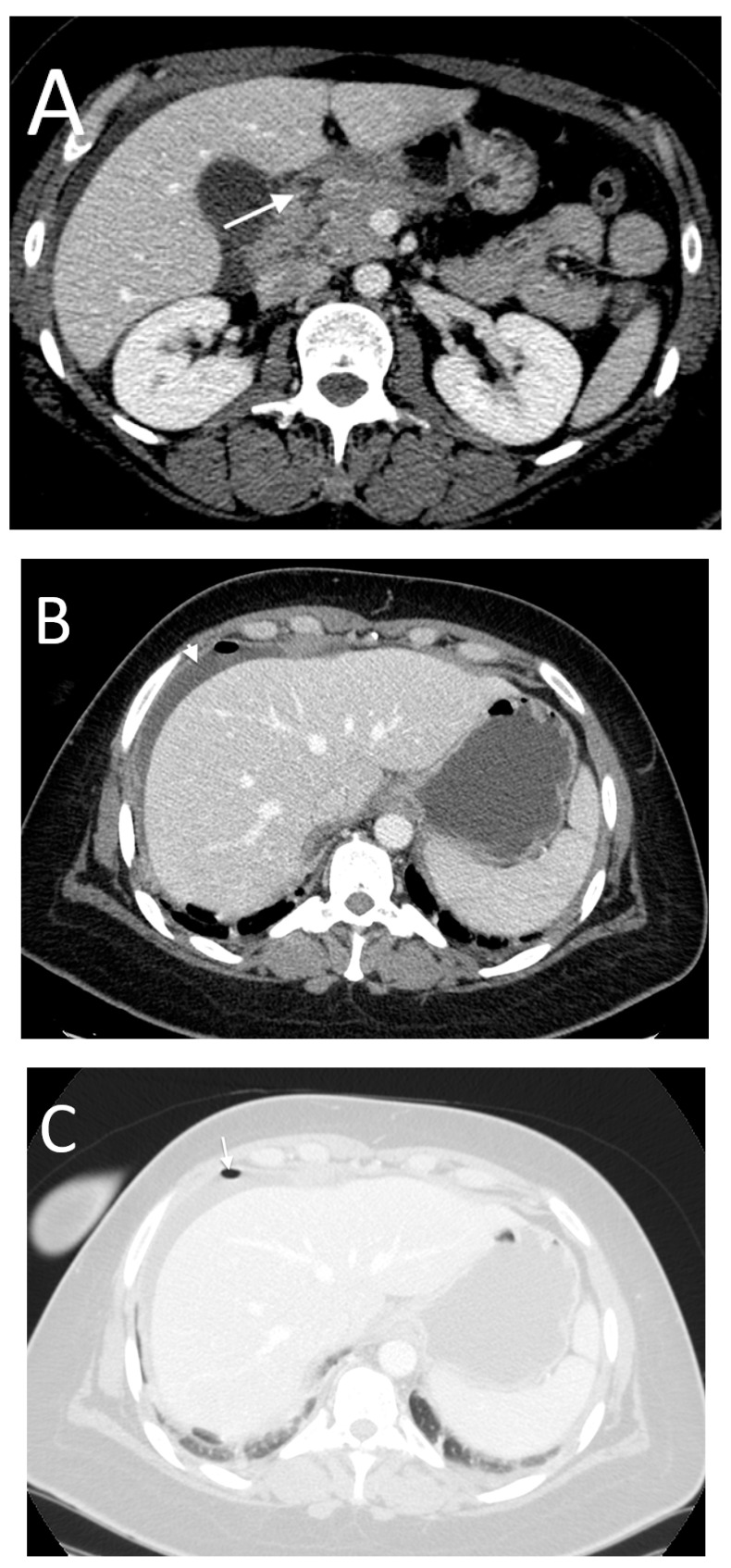
A 45-year-old female with perforation of the anterior gastric wall. In this case, we found a defect of continuity (arrow in (**A**)) at the site of perforation and subdiaphragmatic free fluid (arrowhead in (**B**)) but a very small amount of free air (arrow in (**C**)).

We found a focal wall defect (Figure 7) in 42% of patients with gastric or duodenal perforation but with a PPV of 38%, which could be considered quite with high respect to that found in Groups B–D (PPVs of 24%, 12% and 26%, respectively).

Fat stranding was found in 58% of gastroduodenal perforations in our study, with the highest PPV (35%) close to the perforation site. Lastly, we found distension of the upstream viscera in 61% of cases of perforation of the stomach and duodenum (PPV of 45%), whereas in 58% of patients, we also found a collapse of the downstream loops (PPV of 39%), suggestive of hypotonia of the hollow viscus before the perforation site and spasticity of the visceral tract down to the injured tract.

**Figure 8:**
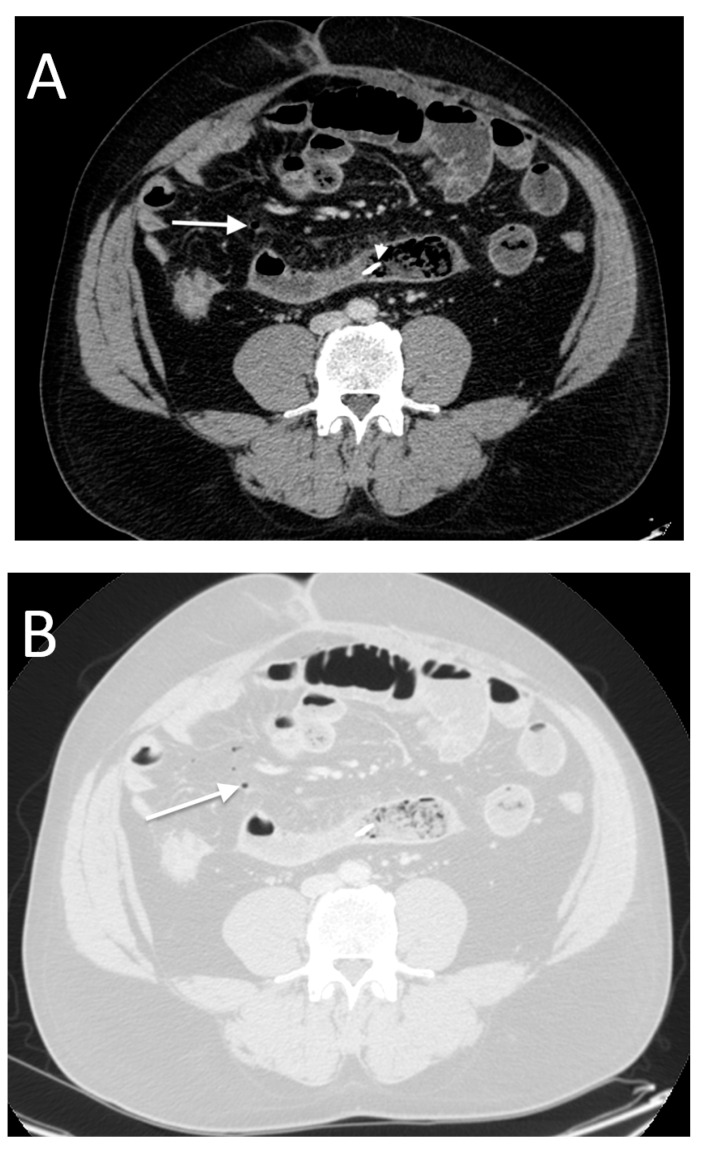
A 54-year-old male with ileal perforation. Only inframesocolic air bubbles (arrow in (**A**,**B**)) were found, with no free peritoneal fluid. Note the hyperdense foreign body in the small bowel loop (arrowhead in (**A**), a chicken bone).

Regarding perforations of the small bowel loops, we found free air in 33% of cases, with a low PPV (10%). This result is in agreement with the literature [11]. In this type of perforation, we found free air bubbles in 61% of cases (Figure 8), and this finding also appears to be in agreement with the literature [11]. However, in Group B of our patient population, we found that small air bubbles located in inframesocolic spaces achieve the highest PPV (50%), and this is an interesting and relevant finding in CT diagnosis of the correct site of perforation.

**Figure 9:**
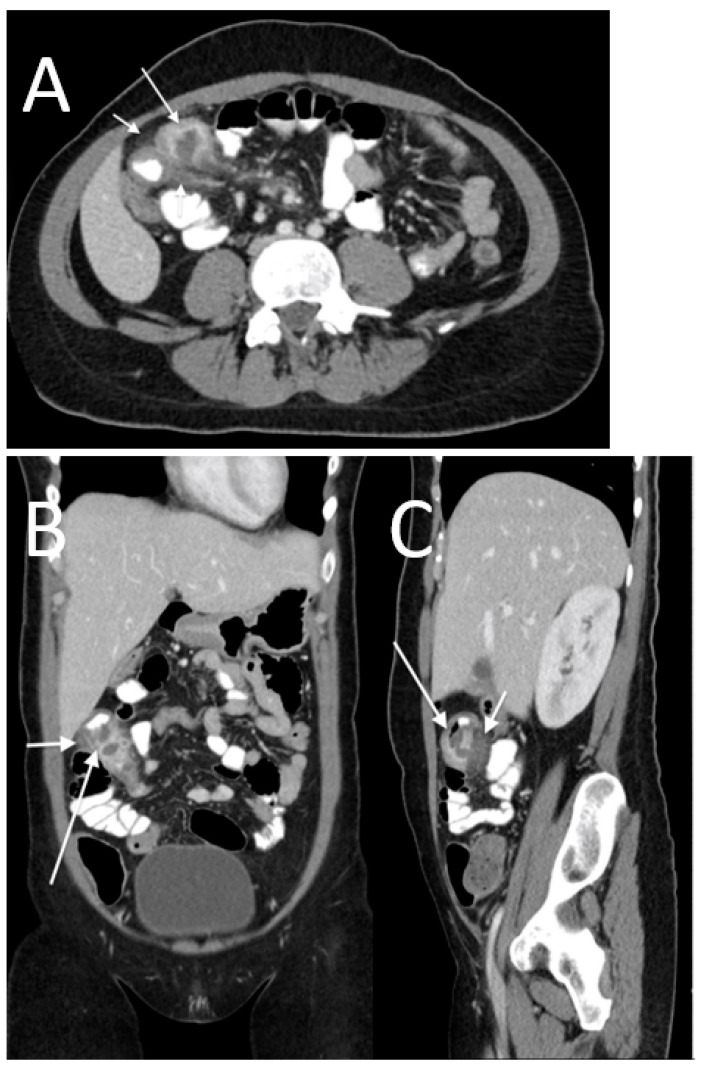
A 35-year-old female with aspergillosis of the ileum and surgical diagnosis of the ischemic ulcerative circumferential lesion at the proximal ileum involving all layers of the ileal wall with concealed perforation. The axial (**A**), coronal (**B**) and sagittal (**C**) images show focal posterior bowel wall disruption with increased enhancement and thickness of the involved bowel segment (long arrows). Surrounding fat stranding and a small amount of fluid are noted (short arrows).

Regarding the evidence of air bubbles close to the perforated loop, it is interesting to note that in our experience, they had a low PPV (PPV of 16%), unlike the known literature (11). We found segmental abnormal wall thickness in patients of Group B and enhancement in 61% and 78% of cases (Figure 9), with a not-so-high PPV (16% and 25%, respectively) regarding the presumptive site of perforation. This finding could be due to the nonspecificity of the bowel wall thickening finding, which can also be found in association with other sites of perforation.

In Group B (small bowel), the upstream loops to the site of perforations were distended in 39% (PPV of 17%) of cases. Air bubbles and fat stranding were found close to the perforation site in 50% of patients but with low PPVs of 16% and 17%. A relatively higher PPV for Group B was found in the wall thickening and enhancement of the downstream loops from the perforation site (22% and 23%, respectively).

**Figure 10:**
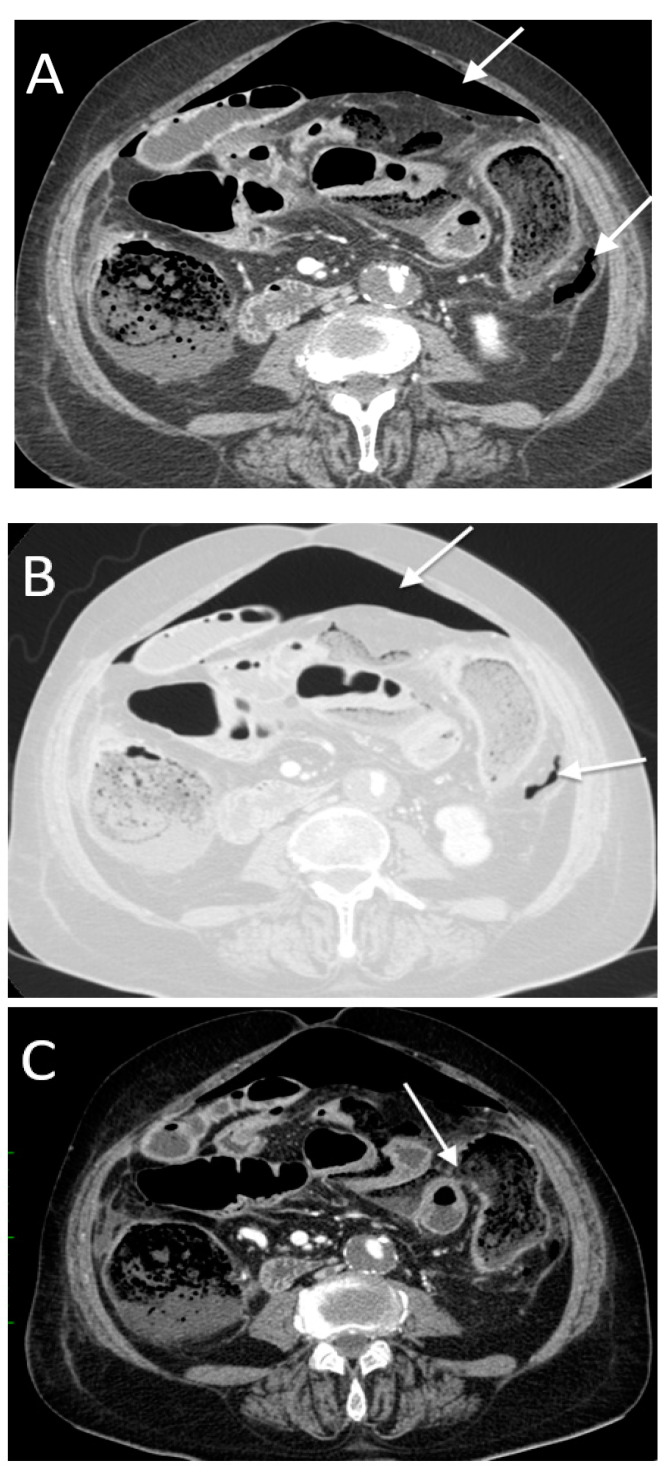

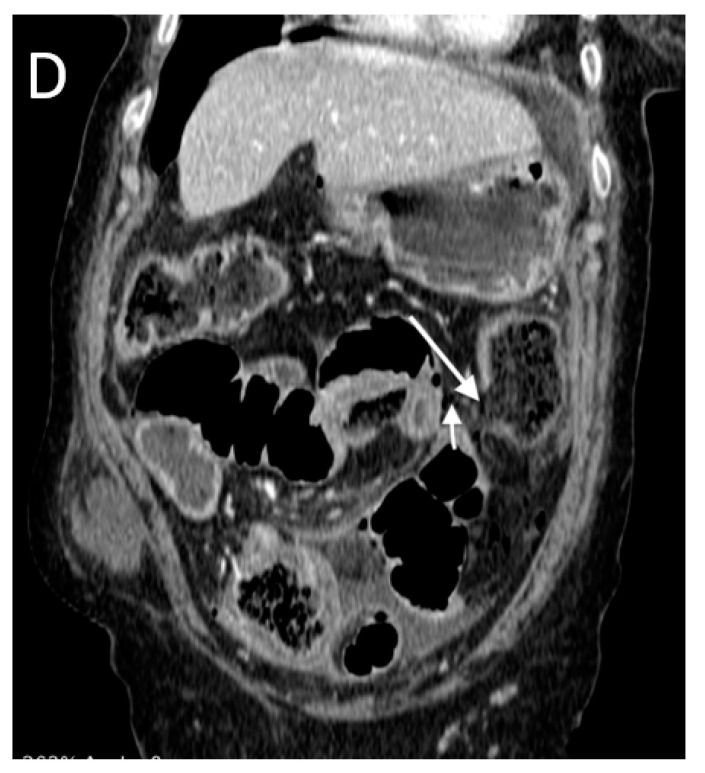
A 67-year-old female with a descending colon perforation. CT images show both supra- and inframesocolic free intraperitoneal air (arrows in (**A**,**B**)). A focal wall defect was identified (arrow in (**C**,**D**)). Note the air bubbles near the perforation site (short arrow in (**D**)) and the reactive segmental wall thickening with the submucosal edema of the ileal loop close to the colonic wall discontinuity.

In colonic perforations from the cecum to the descending colon, free abdominal air was found in 63% of patients, always intraperitoneal, mostly supra- and inframesocolic (50%) (Figure 10).

We found only inframesocolic free air in 20% of cases but with a high PPV (67%). These data are in agreement with those from literature, reporting that the presence of free air only in the inframesocolic space tends to be related to colonic perforation [14]. In our patients, upstream intestinal segments were distended in 50% of cases, and downstream loops collapsed in 50%, but the PPVs were low (19% and 17%, respectively). Interestingly, the fat stranding close to the perforation site was present in 88% of cases (PPV of 27%), and 25% of patients presented fluid collections (PPV of 18%). The combination of free intraperitoneal air and free supramesocolic fluid had a high occurrence in Group C (Figure 2). For sigmoid and rectum perforation (Group D), we found free air mainly inside the peritoneum (83%), especially supra- and inframesocolic (53%), but this latter finding had a lower PPV of 33% compared to the finding related to perforation of the stomach and duodenum (Group A, 38%). In Group D, we did not find free air inframesocolic only, and this finding differs somewhat from that of the literature, reporting that if free gas is present only in the pelvis, usually the site of perforation is related to the colon or the sigmoid colon [14]. For sigmoid perforation, although it was found in 25% of cases, free retroperitoneal air had a high PPV (54%). In fact, a sigmoid diverticulum can perforate into the mesosigmoid, with gas tracking into the retroperitoneum [14]. According to the literature, free retroperitoneal gas, often in the anterior pararenal space, may also be due to perforations during colonoscopy at the level of the posterior wall of the sigmoid of the ascending and descending colon [17].

**Figure 11:**
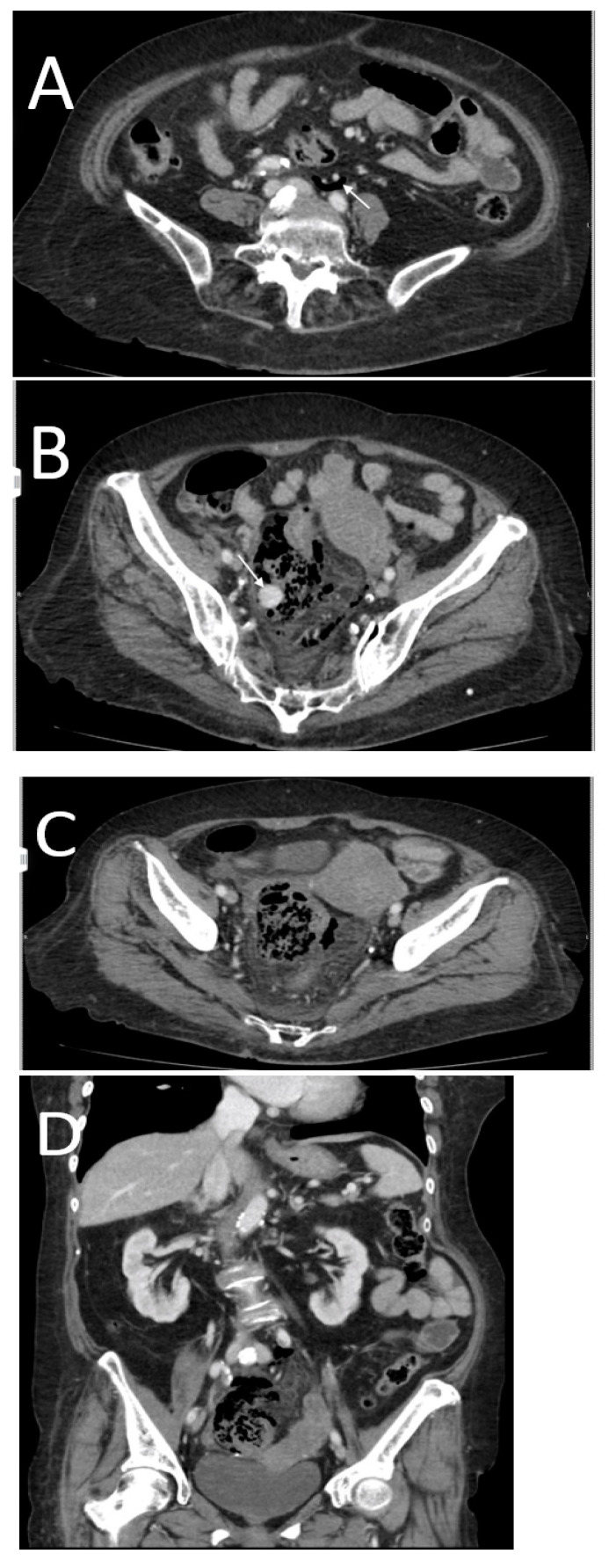

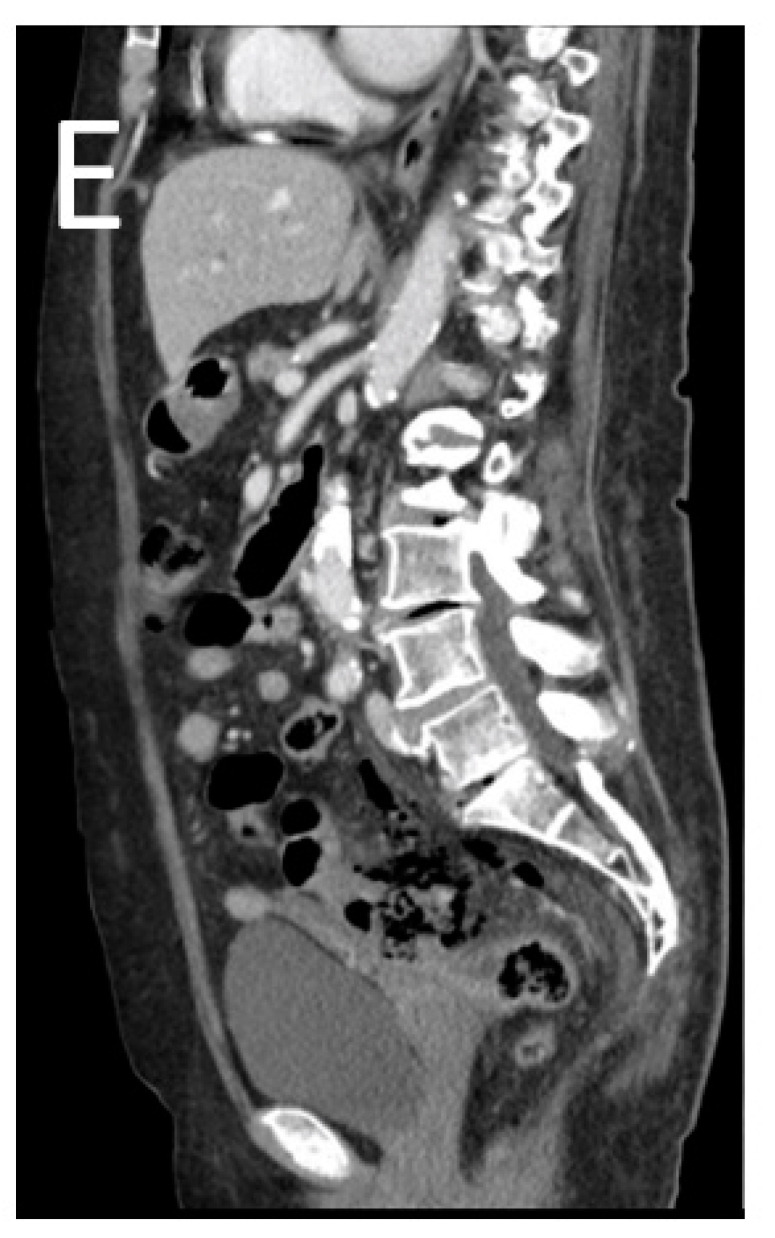
A 69-year-old female with a history of santol seed ingestion. Surgical diagnosis was full thickness of necrosis of the upper rectum with retained santol seed in the submucosal layer. CT shows retroperitoneal free air in the inframesocolic compartment (arrow in (**A**)), round hyperdense opacification in the middle of the fecal-like content adjacent to the involved upper rectum (arrow in (**B**)) and free air close to the involved upper rectum (**C**). Note the focal bowel wall disruption with abnormal thickness and enhancement of the involved upper rectum (**D**,**E**).

A combination of intra- and retroperitoneal free air was found in our study in 22% of cases but with a high PPV of 57%. The evidence of air randomly distributed in the peritoneal and retroperitoneal space could be a challenge in detecting the site of perforation, especially in the presence of extraluminal fecal material. In our experience, we found two cases of stercoral perforation (Figure 11), which is a distinct clinical and pathologic entity [18] caused in most cases by a fecaloma and characterized by the presence of both air and fecal material in the extraluminal space.

**Figure 12:**
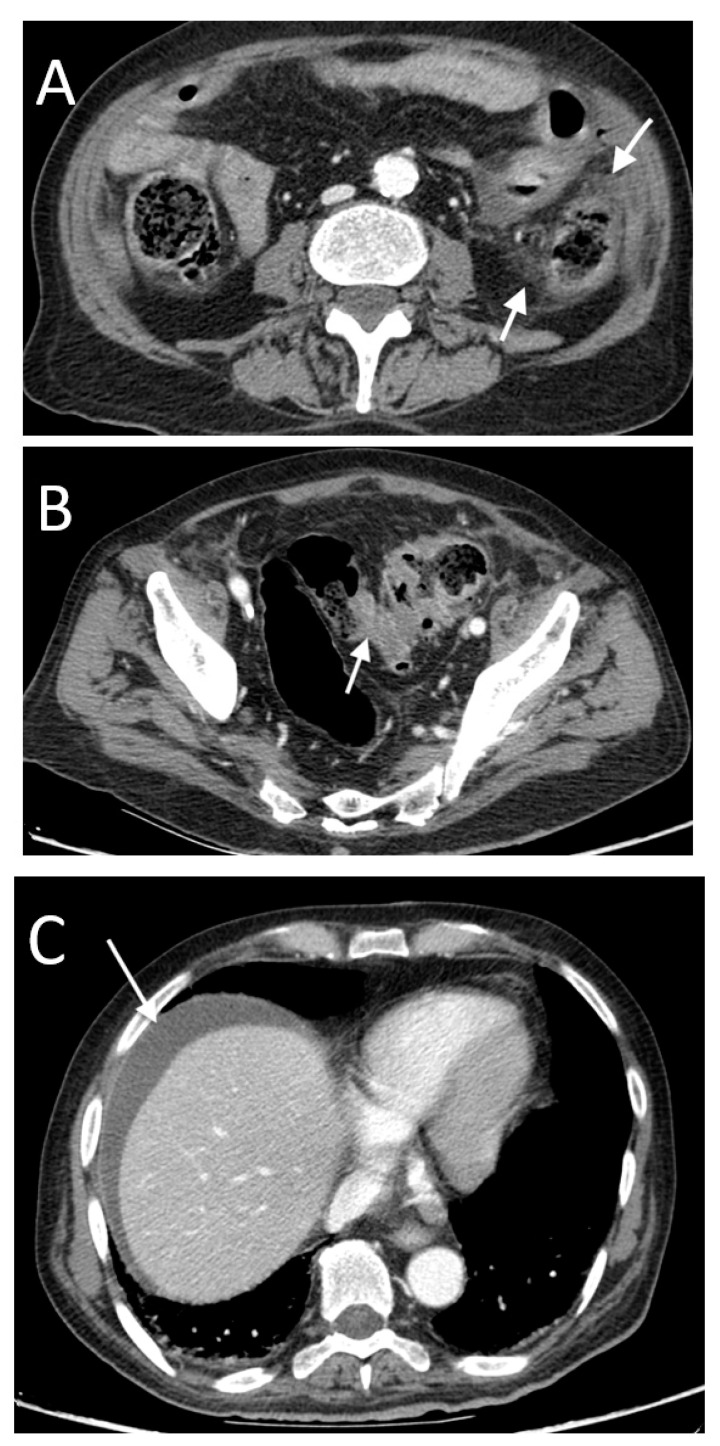
A 77-year-old male with sigmoid colon perforation. Note perivisceral fat stranding and edema surrounding the descending colon (arrows in (**A**)). The perforated segment showed wall edematous thickening and enhancement (arrow in (**B**)). There was no free peritoneal air evidence but perihepatic free fluid (arrow in (**C**)).

The highest PPV of 59% was found in our study for perivisceral fluid collection in 46% of perforations of the sigmoid colon and rectum, followed by intra- and retroperitoneal free air bubbles (30% of cases, PPV of 58%). Additionally, retroperitoneal free fluid had a relatively high PPV of 54% for sigmoid rectal perforations in our study. An interesting finding never investigated before in the literature as per our knowledge is the “perivisceral fat stranding” finding, which, in our case, had the highest PPV of 37% for sigmoid colorectal perforations than other sites and could be helpful to emergency radiologists in diagnosis. For this group of intestinal perforations, in 46% of cases, the fat stranding sign was located at the nearest upstream intestinal segment more than in the exact site of perforation (Figure 12), showing a PPV of 100% (Table 1). Regarding the feature of the upstream and downstream loops in Group D, we did not find any relevant information on luminal distension, but a segmental abnormal wall thickness in the upstream loops (descending colon and proximal sigmoid colon) was noted in 54% of cases.

In the evaluation and reporting of the MDCT examination of patients with suspected gastrointestinal perforations, it is strongly important to be able to detect the cause and site of perforation, being either the type of surgical approach (laparotomy vs. laparoscopy) or a conservative therapy closely depending on this information [1].

In patients with gastroduodenal ulcer perforation, laparoscopy is a very suitable approach for perforations of the anterior or lateral surface, with better clinical outcomes [5,19]. Instead, perforations of the posterior face of the stomach or duodenum could be better managed by regular open surgery [1]. In the surgical literature, small bowel perforations are best treated by laparotomy, and, among colonic perforations, those caused by cancer would be preferably repaired by open surgery [1]. In contrast, management of perforations from sigmoid acute diverticulitis depends on the stage of the disease, as well as the clinical stability of the patient and the experience of the surgical team [1]. In stable patients with radiological evidence of extraluminal air, MDCT findings, including distant versus pericolonic air, extravasation of rectal contrast medium and the presence of an associated abscess, have been reported to be able to predict failure of initial nonoperative management [20]. Colonic diverticula perforation is one of the most common acute abdominal conditions that lead to patients being submitted to emergency CT examination. According to the modified Hinchey classification (Kaiser et al. 2005) [20], acute diverticulitis may be categorized into six stages (0, Ia, Ib, II, III, IV). Most patients with uncomplicated diverticulitis (0—clinical or Ia—pericolic inflammation or phlegmon) are treated conservatively. Patients with an abscess at a pericolonic site (Ib) or in the pelvis or retroperitoneal space (II) are usually treated conservatively or with percutaneous drainage [20]. Conversely, when there is clinical and/or radiological suspicion of generalized purulent (III) or fecal (IV) peritonitis, emergent surgical intervention should be considered, usually in the form of either sigmoid resection and primary anastomosis (with or without proximal diversion) or Hartmann’s procedure [21,22]. Moreover, in the acute emergent setting, bowel distention, as displayed by preoperative CT imaging, may limit the use of laparoscopy due to a small operative space and an increased risk of iatrogenic injuries [23]. Finally, regarding the cecal appendix perforations, preoperative findings of a localized appendiceal perforation with peritonitis could be a reason for switching from an appendectomy by laparoscopy to an open procedure [24].

## 5. Conclusions

Overall, our results appear interesting regarding the MDCT findings according to the site of perforation, adding some more new information to what is already known and has been reported in the radiologic literature. In particular, we did not find only inframesocolic free air in the perforation of the sigmoid colorectal but both supra- and inframesocolic or supramesocolic only. We rarely observed only supramesocolic free fluid in gastroduodenal perforations, and we found low PPVs for air bubbles close to the perforated loop in small bowel perforation (16%) and from the caecum to the descending colon (14%). A high PPV of segmental abnormal wall thickness and enhancement at the exact site of perforation was found in our experience, especially in gastroduodenal perforations (PPVs of 38% and 46%, respectively), whereas a high PPV of fat stranding (37%) was noted in the case of sigmoid colon and rectum perforations next to the upstream segment in 46% of cases with a PPV of 100%. Finally, we found distension of the upstream viscera and collapse of the downstream intestinal segment with respect to the site of perforation in a relevant percentage of patients (especially in perforations of the stomach, duodenum and from the caecum to the descending colon).

Although ours represents a preliminary study on a limited number of patients, these initial data appear interesting to provide new additional information in cases of doubtful MDCT findings that could be used by radiologists in the identification of the gastrointestinal perforation site in order to be able to support surgeons in choosing the most appropriate treatment modality.

## Figures and Tables

**Table 1:** Contingency tables showing the frequencies of findings with respect to each perforation site. Data are presented as frequency and percentages (with 95% confidence interval). INTRA: intraperitoneal; SUPRA: supramesocolic; INFRA: inframesocolic; RETRO: retroperitoneal.

	GROUP A (*n* = 31)		GROUP B (*n* = 18)		GROUP C (*n* = 16)		GROUP D (*n* = 28)	
	*n*	% (95% CI)	*n*	% (95% CI)	*n*	% (95% CI)	*n*	% (95% CI)
FREE AIR(FA)	27	87 (70–96)	6	33 (13–59)	10	62 (35–85)	18	64 (44–81)
FA_INTRA	25	81 (63–93)	4	22 (6–48)	10	62 (35–85)	15	54 (34–72)
FA_SUPRA	16	52 (33–70)	2	11 (1–35)	3	19 (4–46)	7	25 (11–45)
FA_INFRA	0	0 (-)	0	0 (-)	2	13 (2–38)	0	0 (-)
FA_SUPRA+INFRA	9	29 (14–48)	2	11 (1–35)	5	31 (11–59)	8	29 (13–49)
FA_RETRO	5	16 (5–34)	1	6 (0–27)	0	0 (-)	7	25 (11–45)
FA_INTRA+RETRO	3	10 (2–26)	0	0 (-)	0	0 (-)	4	14 (4–33)
AIR BUBBLES(AB)	27	87 (70–96)	11	61 (36–83)	10	62 (35–85)	23	82 (63–94)
AB_INTRA	25	81 (63–93)	10	56 (31–78)	10	62 (35–85)	22	79 (59–92)
AB_SUPRA	15	48 (30–67)	3	17 (4–41)	2	13 (2–38)	7	25 (11–45)
AB_INFRA	0	0 (-)	6	33 (13–59)	2	13 (2–38)	4	14 (4–33)
AB_SUPRA+INFRA	10	32 (17–51)	1	6 (0–27)	6	38 (15–65)	11	39 (22–59)
AB_RETRO	3	10 (2–26)	1	6 (0–27)	3	19 (4–46)	9	14 (41–79)
AB_INTRA+RETRO	1	3 (0–17)	0	0 (-)	4	25 (7–52)	7	25 (11–45)
FREE FLUID(FF)	26	84 (66–95)	13	72 (47–90)	10	62 (35–85)	19	68 (48–84)
FF_INTRA	26	84 (66–95)	13	72 (47–90)	8	50 (25–75)	17	61 (41–79)
FF_SUPRA	2	6 (0–21)	0	0 (-)	2	13 (2–38)	0	0 (-)
FF_INFRA	2	6 (0–21)	2	11 (1–35)	2	13 (2–38)	6	21 (8–41)
FF_SUPRA+INFRA	22	71 (52–86)	11	61 (36–83)	4	25 (7–52)	11	39 (22–59)
FF_RETRO	3	10 (2–26)	1	6 (0–27)	2	13 (2–38)	7	25 (11–45)
FF_INTRA+RETRO	3	10 (2–26)	1	6 (0–27)	2	13 (2–38)	6	21 (8–41)
WALL THICKNESS	26	84 (66–95)	11	61 (36–83)	11	69 (41–89)	21	75 (55–89)
WALL ENHANCEMENT	26	84 (66–95)	14	78 (52–94)	4	25 (7–52)	12	43 (24–63)
WALL DEFECT	13	42 (25–61)	8	44 (22–69)	4	25 (7–52)	9	32 (16–52)
	***n***	**% (95% CI)**	***n***	**% (95% CI)**	***n***	**% (95% CI)**	***n***	**% (95% CI)**
FAT STRANDING	18	58 (39–75)	9	50 (26–74)	14	88 (62–98)	24	86 (67–96)
NEXT TO PERF. LOOP	18	58 (39–75)	9	50 (26–74)	14	88 (62–98)	11	39 (22–59)
NEXT TO UPSTREAM LOOP	0	0 (-)	0	0 (-)	0	0 (-)	13	46 (28–66)
FLUID COLLECTIONS	3	10 (2–26)	2	11 (1–35)	4	25 (7–52)	13	46 (28–66)
AIR BUBBLES CLOSE TO PERF. LOOP	24	77 (59–90)	9	50 (26–74)	8	50 (25–75)	17	61 (41–79)
UPSTREAM LOOPS	***n***	**% (95% CI)**	***n***	**% (95% CI)**	***n***	**% (95% CI)**	***n***	**% (95% CI)**
DISTENSION	19	61 (42–78)	7	39 (17–64)	8	50 (25–75)	8	29 (13–49)
COLLAPSE	4	13 (4–30)	5	28 (10–53)	2	13 (2–38)	13	46 (28–66)
NORMAL	8	26 (12–45)	6	33 (13–59)	6	38 (15–65)	7	25 (11–45)
WALL THICKNESS	12	39 (22–58)	5	28 (10–53)	4	25 (7–52)	15	54 (34–72)
ENHANCEMENT+	14	45 (27–64)	7	39 (17–64)	5	31 (11–59)	7	25 (11–45)
DOWNSTREAM LOOPS	***n***	**% (95% CI)**	***n***	**% (95% CI)**	***n***	**% (95% CI)**	***n***	**% (95% CI)**
DISTENSION	6	19 (7–37)	3	17 (4–41)	3	19 (4–46)	7	25 (11–45)
COLLAPSE	18	58 (39–75)	9	50 (26–74)	8	50 (25–75)	11	39 (22–59)
NORMAL	7	23 (10–41)	6	33 (13–59)	5	31 (11–59)	10	36 (19–56)
WALL THICKNESS	12	39 (22–58)	7	39 (17–64)	2	13 (2–38)	11	39 (22–59)
ENHANCEMENT+	13	42 (25–61)	7	39 (17–64)	3	19 (4–46)	7	25 (11–45)

**Table 2:** Positive predictive values (PPVs) of each finding by perforation site.

	**GROUP A** **PPV (%)**	**GROUP B** **PPV (%)**	**GROUP C** **PPV (%)**	**GROUP D** **PPV (%)**
FREE AIR(FA)	44	10	16	30
FA_INTRA	46	7	19	28
FA_SUPRA	57	7	11	25
FA_INFRA	0	0	100	0
FA_SUPRA+INFRA	38	8	21	33
FA_RETRO	38	8	0	54
FA_INTRA+RETRO	43	0	0	57
AIR BUBBLES(AB)	38	15	14	32
AB_INTRA	37	15	15	33
AB_SUPRA	56	11	7	26
AB_INFRA	0	50	17	33
AB_SUPRA+INFRA	36	4	21	39
AB_RETRO	19	6	19	56
AB_INTRA+RETRO	8	0	33	58
FREE FLUID(FF)	38	19	15	28
FF_INTRA	41	20	13	27
FF_SUPRA	50	0	50	0
FF_INFRA	17	17	17	50
FF_SUPRA+INFRA	46	23	8	23
FF_RETRO	23	8	15	54
FF_INTRA+RETRO	25	8	17	50
WALL THICKNESS	38	16	16	30
WALL ENHANCEMENT	46	25	7	21
WALL DEFECT	38	24	12	26
	**GROUP A** **PPV (%)**	**GROUP B** **PPV (%)**	**GROUP C** **PPV (%)**	**GROUP D** **PPV (%)**
FAT STRANDING	28	14	22	37
NEXT TO PERF. LOOP	35	17	27	21
NEXT TO UPSTREAM LOOP	0	0	0	100
FLUID COLLECTIONS	14	9	18	59
AIR BUBBLES CLOSE TO PERF. LOOP	41	16	14	29
UPSTREAM LOOPS	**GROUP A** **PPV (%)**	**GROUP B** **PPV (%)**	**GROUP C** **PPV (%)**	**GROUP D** **PPV (%)**
DISTENSION	45	17	19	19
COLLAPSE	17	21	8	54
NORMAL	30	22	22	26
WALL THICKNESS	33	14	11	42
ENHANCEMENT+	42	21	15	21
DOWNSTREAM LOOPS	**GROUP A** **PPV (%)**	**GROUP B** **PPV (%)**	**GROUP C** **PPV (%)**	**GROUP D** **PPV (%)**
DISTENSION	32	16	16	37
COLLAPSE	39	20	17	24
NORMAL	25	21	18	36
WALL THICKNESS	38	22	6	34
ENHANCEMENT+	43	23	10	23

## Data Availability

Not applicable.
